# Supplementary material for: Dual-Template Magnetic Molecularly Imprinted Polymer for Simultaneous Determination of Spot Urine Metanephrines and 3-Methoxytyramine for the Diagnosis of Pheochromocytomas and Paragangliomas
Source: Molecules. 2022 May 30;27(11):3520. doi: 10.3390/molecules27113520 (PMC9182035; doi:10.3390/molecules27113520)
Supplement: Supplementary file 1 [file molecules-27-03520-s001.zip › molecules-1745063-supplementary.pdf]

Table S1. The effect of different ratios of the functional monomer to the template molecule on the adsorption efficiency of synthesized MMIP ($\bar{x}$±*s*, *n* = 3).

Functional monomer (mmol)	Template (mmol)	E_A (%)		
		NMN	MN	3-MT
1.000	0.100	91.4±0.5	83.5±0.4	94.4±0.2
1.000	0.050	91.3±0.1	89.4±0.2	94.6±0.0
1.000	0.025	87.6±0.1	74.9±0.3	89.9±0.4

Table S2. The effect of different ratios of the functional monomer and the cross-linking reagent on the adsorption efficiency of synthesized MMIP ($\bar{x}$±*s*, *n* = 3).

Functional monomer (mmol)	Crosslinking reagent (mmol)	E_A (%)		
		NMN	MN	3-MT
1.000	0.500	79.1±0.3	56.4±0.2	84.0±0.6
1.000	1.000	87.6±0.1	74.9±0.3	89.9±0.4
1.000	2.000	83.4±0.5	71.4±0.8	87.0±0.3
1.000	4.000	77.5±0.9	70.6±0.8	83.7±0.8
1.000	8.000	70.5±0.2	62.9±0.9	76.0±0.8

Table S3. The precision of MMIP (n = 5).

	Intra-assay (Peak area)		Inter-assay (Peak area)	
	$\bar{x}\pm s$	CV (%)	$\bar{x}\pm s$	CV (%)
Normal Group				
NMN	1.3±0.1	4.9	1.3±0.1	6.3
MN	17.8±0.8	4.7	17.7±0.5	2.8
3-MT	35.9±0.6	1.7	38.6±1.4	3.7
PPGL Group				
NMN	57.4±0.9	1.6	56.3±2.1	3.7
MN	246.5±9.1	3.7	251.2±10.9	4.3
3-MT	66.3±1.8	2.8	65.1±2.1	3.2

Table S4. The reuse times of MMIP (n = 7).

	NMN (Peak area)		MN (Peak area)		3-MT (Peak area)	
	$\bar{x}\pm s$	CV (%)	$\bar{x}\pm s$	CV (%)	$\bar{x}\pm s$	CV (%)
PPGL	56.3±1.9	3.4	247.2±11.3	4.6	65.5±2.1	3.2
Normal	1.3±0.1	5.5	17.7±0.7	4.0	36.9±1.8	5.0

Table S5. The stability of MMIP (*n* = 3).

	NMN (Peak area)		MN (Peak area)		3-MT (Peak area)	
Days	$\bar{x}\pm s$	Reduction (%)	$\bar{x}\pm s$	Reduction (%)	$\bar{x}\pm s$	Reduction (%)
1	122.1±0.7	-	144.2±3.6	-	171.1±3.5	-
14	115.8±2.7	5.2	143.3±4.4	0.7	166.5±4.5	2.7
28	100.5±0.6	17.6	127.4±2.0	11.6	139.3±4.8	18.6

Table S6. Calibration curves for NMN, MN and 3-MT in urine samples (*n* = 3).

Analyte	Calibration curves	R^2	LOD (μg/L)	LOQ (μg/L)	Linearity (μg/L)
NMN	Y=0.4053 *X*+4.6132	0.9992	4.2	15.3	25.0-1000.0
MN	Y=0.5337 *X*+1.7614	0.9997	9.6	24.2	25.0-1000.0
3-MT	Y=0.6953 *X*-5.3064	0.9994	7.7	21.3	25.0-1000.0

Table S7. Recoveries of the assay ($\bar{x}$±*s*, *n* = 3).

Analyte	Added concentration (μg/L)	Recovery (%)	CV (%)
NMN	50.0	105.5±6.8	6.4
	250.0	93.2±1.8	1.9
	800.0	112.8±7.2	6.4
MN	50.0	97.1±1.9	2.0
	250.0	99.3±0.7	0.7
	800.0	96.3±1.3	1.4
3-MT	50.0	99.0±1.0	1.0
	250.0	100.5±0.4	0.4
	800.0	101.7±0.4	0.3

Table S8. The spot urine A_{MNs}/A_{Cr} results of PPGLs group and control group pretreated by SPE and d-SPE.

SPE	PPGLs group (n=18)	Control group （n=22）
A_{NMN}/A_{Cr}	6046.6 [a] (1523.8-16133.5)	1020.2 (276.8-2791.4)
A_{MN}/A_{Cr}	192.5 [a] (1.4-2937.4)	25.9 (6.7-173.6)
$A_{3\text{-}MT}/A_{Cr}$	507.7 (3.9-15515.7)	501.0 (113.3-5544.5)
d-SPE		
A_{NMN}/A_{Cr}	2527.3 [a] (8.4-7952.9)	557.8 (143.4-1191.7)
A_{MN}/A_{Cr}	440.5 [a] (1.7-4060.7)	33.7 (8.7-226.0)
$A_{3\text{-}MT}/A_{Cr}$	244.3 (4.5-17074.9)	352.3 (133.3-1403.7)

Annotation: The results were shown as median (min, max); the comparation of PPGLs group and the control groups were conducted by paired sample t-test; a means compared with the control group (P<0.05).

Table S9. The sensitivity and specificity of plasma MNs and spot urine free MNs for the diagnosis of PPGLs (n=40).

	Cut-off	Sensitivity (%)	Specificity (%)	AUC
Plasma				
NMN(μg/L)	123.1	100.0	90.9	0.975
MN(μg/L)	130.6	72.2	100.0	0.773
Urine				
A_{NMN}/A_{Cr}	1347.1	83.3	100.0	0.990
A_{MN}/A_{Cr}	90.2	83.3	90.9	0.821

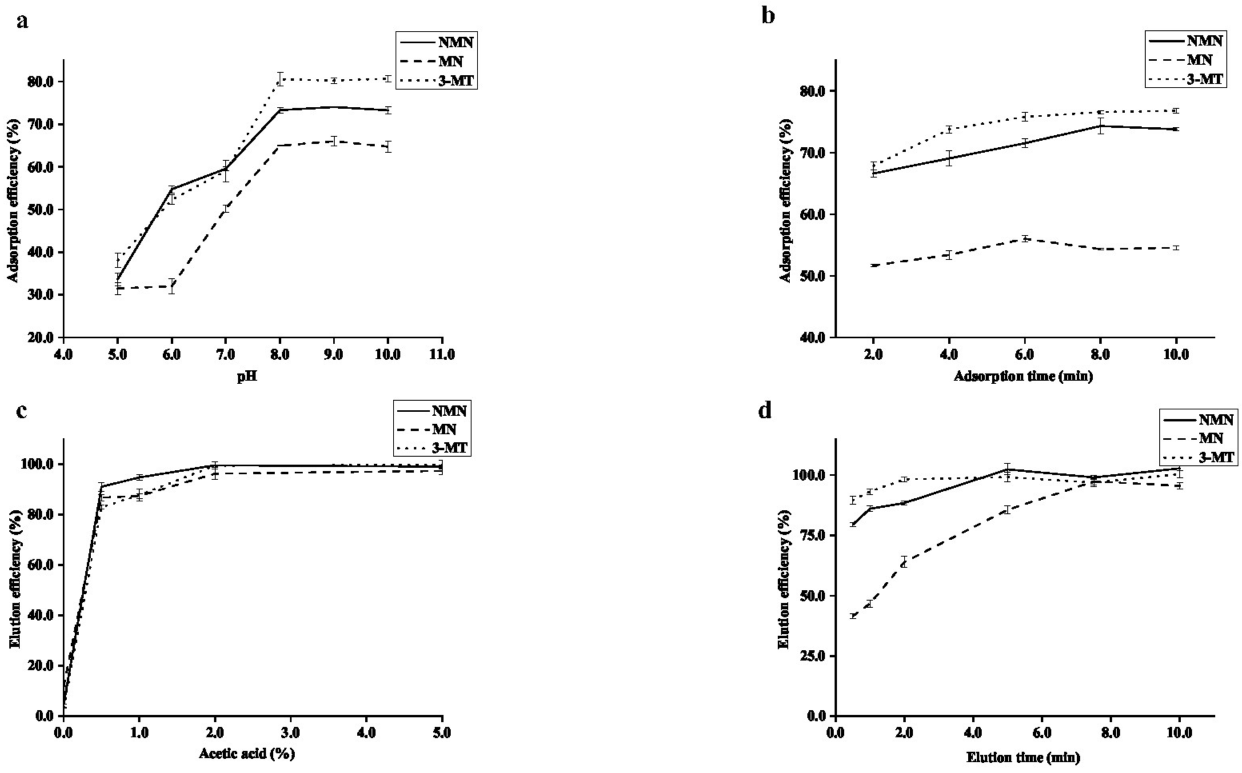

Figure S1. The optimization of experimental conditions for d-SPE.

(a. pH; b. adsorption time; c. concentration of acetic acid; d. elution time) ($\bar{x}$±s, n = 3)
